# Geographical traceability of bacteria based on a systematic stable isotope analysis method

**DOI:** 10.1007/s00253-025-13704-x

**Published:** 2026-01-19

**Authors:** Wei Wang, Bichun Zhao, Zhuotong Cai, Zhaowei Jie, Lin Feng, Can Hu, Hongling Guo, Yajun Li, Xianhe Deng, Jun Zhu, Hongcheng Mei, Jian Ye

**Affiliations:** 1 People’s Public Security University of China, Beijing, 100038 China; 2 Institute of Forensic Science, Ministry of Public Security of China, Beijing, 100038 China; 3 Department of Public Security of Shanxi Province, Taiyuan, 030001 Shanxi China; 4 Beijing University of Chinese Medicine, Beijing, 102488 China

**Keywords:** Bacteria, Geographical traceability, Stable isotope analysis method, Isotope fractionation pattern, Multi-isotope combination discriminant model

## Abstract

**Abstract:**

The outbreak of certain bacteria can trigger severe diseases, even posing a threat to public safety, leading to significant social panic and economic losses. Thus, tracing the origin of bacteria is of great significance. Stable isotope analysis technology offers a new way to determine the geographical information of bacteria, yet related research still fails to meet the application requirements of this technology in practical cases of bacterial traceability. In this study, a systematic stable isotope analysis method for bacteria and their culture conditions, based on practical geographical environments, was established for the first time. *Escherichia coli* and *Staphylococcus aureus* were cultured with water from five regions and different culture media, and the stable isotope ratios of H/O/C/N in the two bacteria and the culture media were measured to explore the relationship between bacteria and their cultivation site. The results showed that there were linear relationships between the hydrogen and oxygen stable isotopes of the two bacteria and the culture water. The combined discriminant model constructed using multi-isotope (H/O/C/N) characteristics achieved a 100% accuracy rate in identifying the types of culture media. These results indicate that research on the isotope association between bacteria and their culture water can be used to infer the cultivation region, and the specific source of bacteria can be further inferred through the multi-isotope combination discriminant model. This study can provide a relatively complete research idea for bacterial geographical traceability research, and improve the efficiency and accuracy of bacterial traceability work in practical investigations.

**Key points:**

- *Established a systematic stable isotope analysis method for bacteria.*
- *E. coli and S. aureus have linear H/O isotope correlations with culture water.*
- *Multi-isotope discriminant model achieves 100% accuracy in identifying culture media.*

**Supplementary Information:**

The online version contains supplementary material available at 10.1007/s00253-025-13704-x.

## Introduction

Prokaryotes are ubiquitous on Earth, primarily inhabiting the open ocean, soils, and both oceanic and terrestrial subsurfaces (Whitman et al. 1998). As common prokaryotes, bacteria play a crucial role in shaping the Earth’s ecosystems. Among the diverse range of bacteria, the outbreak of certain bacterial species can trigger severe diseases and even pose a threat to public safety (Jernigan et al. 2002; Zacchia and Schmitt 2019). Typical cases include the anthrax spore mail attacks in the USA in 2001 (Gautier 2023) and the global cholera pandemics (Kanungo et al. 2022), which have caused great social panic and economic losses. To effectively resolve such incidents and prevent them from happening again, researchers have established various methods to trace the origin and transmission routes of bacteria. Currently, the methods for tracing the origin of bacteria mainly rely on genetic information (such as genome sequencing), phenotypic traits, and epidemiological investigations (Ma et al. 2023; Payne et al. 2023; Yu et al. 2023). However, they are not capable of tracing the cultivation sites of bacteria.

Stable isotope analysis technology offers a new approach for studying the traceability of bacteria. Stable isotopes, which refer to elements having the same atomic number and chemical properties, possess different atomic masses and physical properties. Researchers introduce isotope abundance to describe the relative abundance of elements (the proportion of the number of atoms of a specific isotope to the total number of atoms of the element) (Hu et al. 2025; Ma et al. 2025). Since the natural abundance of most heavy isotopes, such as those of hydrogen and oxygen, is extremely low, in practical analysis, the relative isotope ratio *R* is more frequently employed to describe abundance (*R* = heavy isotope abundance/light isotope abundance). To characterize differences in stable isotopes, the scientific community uses the isotope delta (*δ*) value. For an element *E* in a substance *P*, *δ* is defined as the relative difference in isotope ratios between the sample and an international standard (Std): *δ*_Std_ (^*i/j*^*E*, *P*) = *R* (^*i/j*^*E*, *P*)/*R* (^*i/j*^*E*, Std) − 1 (Skrzypek et al. 2022). Currently, 274 stable isotopes have been identified, including ^2^H and ^1^H, ^18^O and ^16^O, ^13^C and ^12^C, and ^15^N and ^14^N. The abundances of hydrogen and oxygen stable isotopes exhibit significant geographical differences, while the carbon and nitrogen stable isotopes are affected by plant distribution and also show regional differences on a large scale, forming a geographical spatial pattern characterized by stable isotope abundances (Amundson et al. 2003; Li et al. 2025; Still et al. 2003).

Bacteria rely on nutrients and water for growth, leading to unique stable isotope ratio characteristics in their cells (Kreuzer-Martin et al. 2005; Kreuzer-Martin and Jarman 2007). These characteristics are directly related to the growth environment. Previous studies, by artificially controlling experimental conditions, have verified that the isotopic composition of bacteria is correlated with the stable isotope ratios of their growth media and water (Horita and Vass 2003; Kreuzer-Martin et al. 2004). Nevertheless, the growth conditions of bacteria in these studies were not designed based on real-world geographical environments. Thus, their research findings are difficult to apply directly in real-life cases. In addition, it is challenging to conduct similar research on other bacteria. Moreover, through a literature review, we found that there is currently no research on systematic methods for the stable isotope analysis of bacteria and their culture systems, which involve the investigation of instrumental analysis conditions such as bacterial incubation time, washing cycles, and temperature settings for TC-EA and EA. Therefore, the aim of this study is to establish a set of stable isotope analysis methods based on practical geographical environments, with the advantages of economy and operational simplicity, and to employ these methods to investigate the relationship between bacteria and their culture water and media, ultimately enabling the inference of the bacteria’s cultivation site.

In the practical investigation of bacterial traceability, the analysis methods and experimental procedures established in this study can be utilized as a reference. By studying the relationship between bacteria and their culture water and culture media, it becomes feasible to determine the cultivation region of the bacteria. This not only improves the efficiency and accuracy of bacterial traceability but also offers valuable insights for relevant researchers in this field.

## Materials and methods

### Materials

#### Bacterial strains

The experiment utilized *Escherichia coli* (*E. coli*, CICC® 10389) and *Staphylococcus aureus* (*S. aureus*, CICC® 21600), purchased from the China Center of Industrial Culture Collection (CICC).

#### Culture media and water

LB medium (Luria-Bertani medium) was collected from three different manufacturers. Each formulation consisted of tryptone (10 g/L), yeast extract (5 g/L), and sodium chloride (10 g/L), with the pH adjusted to 7.4. The manufacturers were Qingdao Hope Bio-technology Company Limited (batch number, 20230710), Qingdao Rishui Bio-technology Company Limited (batch number, 20230803), and Guangdong Huankai Microbial Technology Company Limited (batch number, 230602A40).

TSB medium (tryptic soy broth medium) was obtained from three different manufacturers. Each formulation consisted of tryptone (17 g/L), soybean peptone (3 g/L), sodium chloride (5 g/L), dipotassium hydrogen phosphate (2.5 g/L), and glucose (2.5 g/L), with the pH adjusted to 7.3. The manufacturers were Qingdao Hope Bio-technology Company Limited (batch number, 20230523), Qingdao Rishui Bio-technology Company Limited (batch number, 20230707), and Beijing SanYao Technology Company Limited (batch number, 2305052).

NB medium (nutrient broth medium) was collected from eight different manufacturers. Each formulation consisted of peptone (10 g/L), beef extract powder (3 g/L), and sodium chloride (5 g/L), with the pH adjusted to 7.2. The manufacturers were Beijing Aoboxing Bio-technology Company Limited (batch number, 20230522), Qingdao Rishui Bio-technology Company Limited (batch number, 20230401), Beijing Land Bridge Technology Company Limited (batch number, 230216), Shanghai Biowell Microbial Technology Company Limited (batch number, 20230613), Beijing Hongrun Baoshun Technology Company Limited (batch number, 230515), Qingdao Hope Bio-technology Company Limited (batch number, 20230322), Guangdong Huankai Microbial Technology Company Limited (batch number, 230217A20), and Beijing SanYao Technology Company Limited (batch number, 221102).

#### Water samples

Water samples were collected from China Center of Industrial Culture Collection (Beijing, China), Tongji University (Shanghai, China), Sun Yat-sen University (Guangzhou, China), Chongqing Medical University (Chongqing, China), and Jilin University (Changchun, China). All samples were prepared using a water purification system (Merck Milli-Q) in the laboratory. They were transported and stored at 4 °C in the dark. Before use, all samples were sterilized (121 °C, 103.4 kPa, 15 min).

#### Instruments and reagents

Elemental analyzer, Flash EA 2000, Thermo Scientific (USA); stable isotope ratio mass spectrometer, 253 Plus, Thermo Scientific (USA); continuous flow interface, ConFlo Ⅳ, Thermo Scientific (USA); zero-blank autosampler, Thermo Scientific (USA); wavelength-scanned cavity ring-down spectrometer (WS-CRDS), L2130i, Picarro (USA); electric thermostatic drying oven, Model 202-0AB, Beijing Zhongxing Weiye Instrument Co., Ltd. (China); electronic balance, XPR 2, Mettler Toledo (Switzerland); experimental water, prepared using a Merck Milli-Q (Germany). Tin and silver cups, Santis (Switzerland).

#### Stable isotope reference materials (RMs)

CBS (*δ*^2^H_VSMOW-SLAP_ = −157 ± 0.9‰, *δ*^18^O_VSMOW-SLAP_ = 3.8 ± 0.3‰), USGS40 (*δ*^13^C_VPDB-LSVEC_ = −26.39 ± 0.04‰, *δ*^15^N_Air_ = −4.52 ± 0.06‰), USGS41a (*δ*^13^C_VPDB-LSVEC_ = 36.55 ± 0.08‰, *δ*^15^N_Air_ = 47.55 ± 0.15‰), USGS42 (*δ*^2^H_VSMOW_ = −72.9 ± 2.2‰, *δ*^18^O_VSMOW_ = 8.56 ± 0.10‰, *δ*^13^C_VPDB-LSVEC_ = −21.09 ± 0.10‰, *δ*^15^N_Air_ = 8.05 ± 0.10‰), USGS43 (*δ*^2^H_VSMOW_ = −44.4 ± 2.0‰, *δ*^18^O_VSMOW_ = 14.11 ± 0.10‰), VSMOW2 (*δ*^2^H_VSMOW-SLAP_ = 0.0 ± 0.4‰, *δ*^18^O_VSMOW-SLAP_ = 0.0 ± 0.04‰), GISP (*δ*^2^H_VSMOW-SLAP_ = −189.7 ± 1.0‰, *δ*^18^O_VSMOW-SLAP_ = −24.78 ± 0.09‰), SLAP2 (*δ*^2^H_VSMOW-SLAP_ = −427.5 ± 0.4‰, *δ*^18^O_VSMOW-SLAP_ = −55.50 ± 0.04‰). All the above reference materials have standard uncertainty (*k* = 1). The reference materials were purchased from Reston Stable Isotope Laboratory (USA).

## Methods

### Preparation of bacterial powder samples

According to established protocols from the literature (Huang et al. 2006; Welman and Maddox 2003), the bacterial freeze-dried powder was prepared as follows. A 1-mL aliquot of frozen stock (approximately 10^7^–10^8^ cells) of *E. coli* or *S. aureus* was separately inoculated in triplicate into 200 mL of LB, TSB, and NB liquid media. The cultures were incubated in shake flasks at 36 ± 1 °C and 200 rpm for 19 h (This duration was selected based on a systematic study of “Bacterial Incubation Time” to ensure the stability of the bacterial stable isotope ratios). Bacterial growth data was assessed in preliminary experiments (growth curves were constructed by measuring the OD620 every 2 h, as shown in Fig. S3 and S4). Subsequently, the bacterial suspensions were centrifuged at 8000 rpm for 10 min to pellet the cells. After discarding the supernatant, the pellets were resuspended and washed seven times with sterile water prepared using source water from the Beijing region. The harvested wet bacterial paste was lyophilized for 16 h at 0.470 mbar using a Christ Gamma 1–16 LSC freeze dryer. Finally, the dried powder was sterilized by dry heat at 160 °C for 2 h in a CS101-3D electric blast drying oven.

### Stable isotope sample preparation

Based on previous research findings and the optimized operational procedures developed by our group (Hao et al. 2023; Xin-long et al. 2020; Zi-Yang et al. 2022), 250 ± 1 μg of bacterial powder, culture medium, and stable isotope reference materials were weighed (Electronic Balance: XPR 2, Mettler Toledo). They were then encapsulated in tin capsules (Sn cups). Subsequently, all samples were used for carbon and nitrogen isotope delta analyses via an elemental analyzer (EA, combustion) coupled to a stable isotope ratio mass spectrometer. Simultaneously, bacterial powder, culture medium, and stable isotope reference materials were dried at 80℃ for 2 h, equilibrated in a desiccator for 1 week, and then weighed (250 ± 1 μg) into silver capsules (Ag cups), which were used for hydrogen and oxygen isotope delta analyses via a high-temperature conversion elemental analyzer (TC-EA, pyrolysis) coupled to the stable isotope ratio mass spectrometer.

### Instrumental analysis conditions

The TC-EA was configured with a pyrolysis furnace at 1350 °C and a chromatography column at 85 °C, while the EA was equipped with a combustion module at 800 °C and a chromatography column at 50 °C (Details of the pyrolysis column and combustion/reduction column compositions are provided in Fig. S1 and S2 of the Supplementary Materials). For H and O stable isotope analysis, the flow rate of the carrier gas (He) was set to 100 mL/min; for C and N stable isotope analysis, it was 50 mL/min.

The WS-CRDS isotopic water analyzer was configured with a vaporizer at 110 °C and operated with high-purity nitrogen (99.999%) as the carrier gas, while it was set to high precision measurement mode with a background water concentration of less than 30 ppm. The injection volume was approximately 1.9 μL, targeting a water concentration of 19,000–21,000 ppm. The syringe was thoroughly washed with NMP (1-methyl-2-pyrrolidinone, 99%) before measurement, and was rinsed once with the sample before each injection.

### Calculation of isotope delta values

For the determination of *δ*^2^H and *δ*^18^O values in samples, RMs including CBS, USGS42, and USGS43 were used to calibrate, establish multi-point linear regression equations (*R*^2^ > 0.999), and calculate the true stable isotope values. For the determination of *δ*^13^C and *δ*^15^N values of samples, RMs such as USGS40, USGS1a, and USGS42 were used to establish multi-point linear regression equations (*R*^2^ > 0.999), calibrate, and derive the true values. During the entire stable isotope measurement process, quality control (QC) samples were analyzed synchronously: one QC sample was inserted after every 10 test samples (CBS for *δ*^2^H/*δ*^18^O determination, USGS40 for *δ*^13^C/*δ*^15^N determination), and all measured *δ* values of QC samples were required to fall within their certified uncertainty ranges. Prior to sample analysis, each calibration RM was measured in triplicate, and the standard deviation (SD) of these replicate measurements was used to modify the linear regression calibration model, thereby reducing the systematic error caused by the RMs themselves. All uncertainties reported in this study represent the SD of replicate sample measurements; each sample was measured nine times (*n* = 9) through three independent parallel preparations (i.e., independent completion of the entire process of lyophilized, sterilized and packaging), with each prepared sample analyzed once on the instrument. Ultimately, nine valid data points were obtained for each sample, based on which the mean and SD were calculated.

For the determination of *δ*^2^H and *δ*^18^O values in pure water collected from five cities, RMs including VSMOW2, GISP, and SLAP2 were used to establish multi-point linear regression equations (*R*^2^ > 0.999), calibrate, and derive the true stable isotope values. During the measurement, QC samples were analyzed synchronously: one QC sample was inserted after every 10 test samples (VSMOW2, GISP, and SLAP2 were randomly interspersed), and all measured *δ* values of QC samples must lie within their certified uncertainty ranges. For each type of water sample from the five cities, three aliquots were prepared, and each aliquot was measured five times (first two injection results discarded). Finally, nine valid data points were obtained for each type of water sample, and the mean and SD were calculated accordingly.

## Results

### Establishment of stable isotope analysis methods for bacteria and their culture systems

**Fig. 1:**
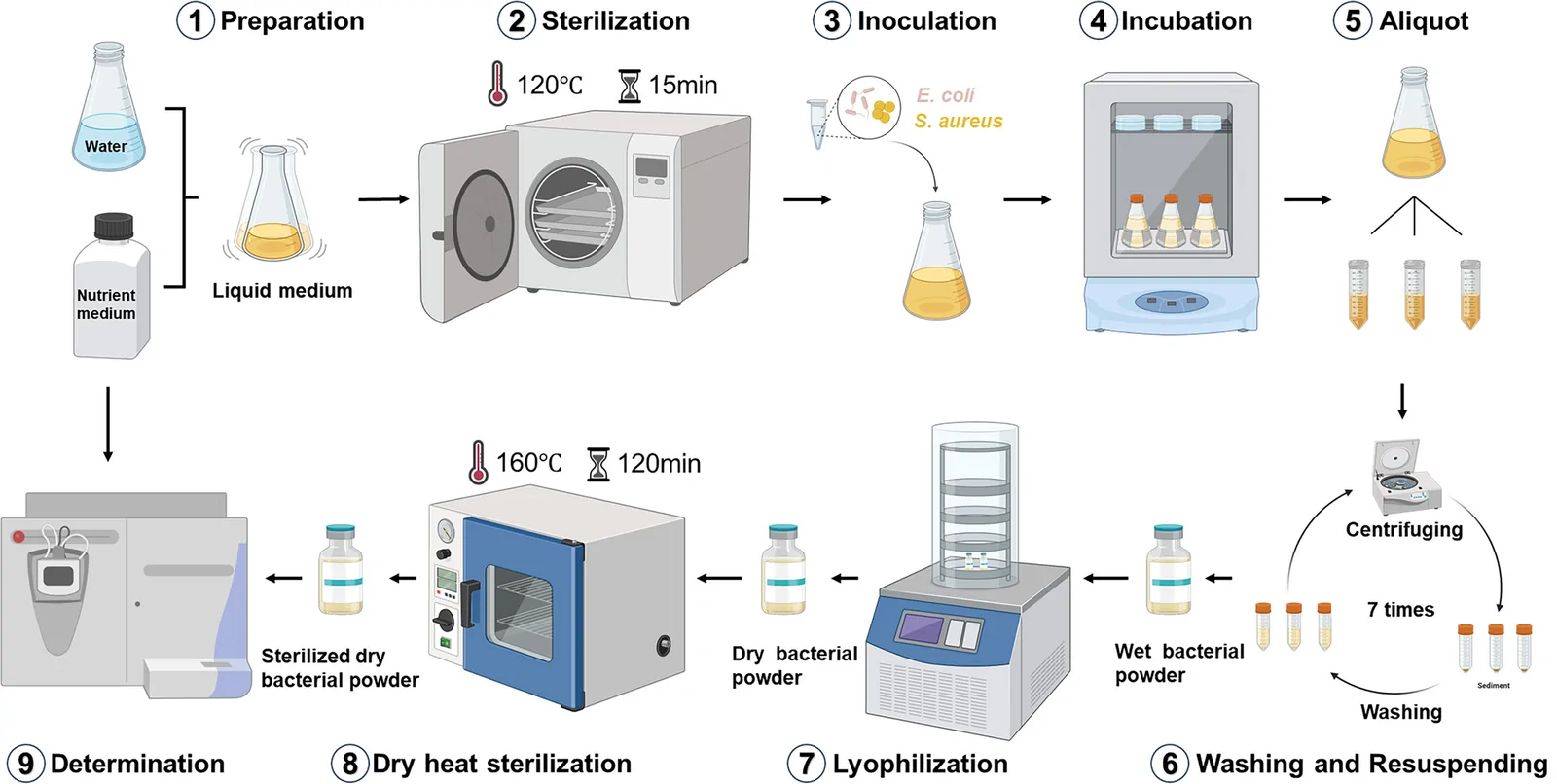
Bacterial cultivation and preparation process of freeze-dried bacterial powder. ⑨ Determination encompasses the stable isotope analysis of both bacterial powder and culture medium

The foundation of bacterial stable isotope analysis lies in the precise determination of stable isotopes in bacteria and their culture systems. To this end, we have drawn a flow chart (Fig. 1) for the preparation of culture medium, bacterial cultivation, bacterial freeze-drying, and preparation of test samples to study the impact of key steps on the stable isotopes of bacteria. It was found that the bacterial incubation time (step 4), washing cycles (step 6), and the key parameters of instrument measurement are most likely to affect the stable isotope characteristics of bacteria. To improve the framework of bacterial stable isotope analysis, we conducted an in-depth exploration of the above three aspects and optimized and refined other steps (data not shown) to ensure the overall accuracy and reliability of the stable isotope analysis method.

### Bacterial incubation time

The impact of incubation time on the stable isotopes of bacteria was determined by setting multiple incubation time points (with incubation times of 10 h, 13 h, 16 h, 19 h, and 22 h) in NB medium (step 4 in Fig. 1). For each time point, three independent biological replicate cultures were performed. From each replicate, three separate aliquots of bacterial powder were prepared for subsequent isotopic measurements. In the preliminary stage, we plotted the growth curves of the two types of bacteria (Fig. S3 and S4). We found that *E. coli* and *S. aureus* entered the stationary phase at 15 h and 16 h, respectively, which served as the basis for setting the incubation time intervals. Bacteria were cultured and bacterial powder was prepared according to the above method (Fig. 1). The *δ*^2^H, *δ*^18^O, *δ*^13^C, and *δ*^15^N values of *E. coli* and *S. aureus* at different incubation times were measured (Fig. S5 and S6). The results showed that for *E. coli*, the *δ*^15^N value increased gradually before 19 h and tended to be stable after 19 h (Fig. S5d). For the *δ*^15^N values of *E. coli*, significant differences were observed at incubation times of 10 h, 13 h, 16 h, and 19 h (*P* < 0.05), whereas no significant differences were detected between 19 and 22 h (*P* > 0.05). Its *δ*^2^H, *δ*^18^O, and *δ*^13^C values remained basically stable across different incubation times (Fig. S5a–c). For *S. aureus*, the *δ*^2^H and *δ*^15^N values increased gradually before 19 h and tended to be stable after 19 h (Fig. S6a,d). For the *δ*^2^H and *δ*^15^N values of *S. aureus*, significant differences were observed at incubation times of 10 h, 13 h, 16 h, and 19 h (*P* < 0.05), whereas no significant differences were detected between 19 and 22 h (*P* > 0.05). Its *δ*^18^O and *δ*^13^C values were basically stable across different incubation times (Fig. S6b,c). These results indicated that using 19 h as the incubation time for both types of bacteria could ensure the stability of their stable isotope measurement results.

### Washing cycles

During cultivation, bacteria produce and secrete various compounds into the culture medium. To prevent these compounds from interfering with the accurate determination of the stable isotope composition of the bacterial powder, we investigated the stable isotope measurement results of bacterial powder under different washing cycles (2–9 times) (step 6 in Fig. 1). The results showed that for *E. coli*, the *δ*^15^N value increased gradually up to seven washing cycles and tended to be stable after seven cycles (Fig. S7d). For the *δ*^15^N values of *E. coli*, significant differences were observed at two to seven washing cycles (*P* < 0.05), whereas no significant differences were detected at seven to nine washing cycles (*P* > 0.05). Its *δ*^2^H, *δ*^18^O, and *δ*^13^C values remained basically stable across different washing cycles (Fig. S7a-c). For *S. aureus*, the *δ*^2^H value decreased gradually up to seven washing cycles and tended to be stable after seven cycles (Fig. S8a). For the *δ*^2^H values of *S. aureus*, significant differences were observed at two to seven washing cycles (*P* < 0.05), whereas no significant differences were detected at seven to nine washing cycles (*P* > 0.05) (Fig. S8a). Its *δ*^18^O, *δ*^13^C, and *δ*^15^N values remained basically stable across different washing cycles (Fig. S8b-d).

The reason for the differences between the two types of bacteria might be that *E. coli* has weak proteolytic activity, and more nitrogen-rich high-molecular-weight hydrophobic polypeptides remain during the cultivation process (Kasal et al. 2023). These polypeptides require prolonged washing for complete removal. In contrast, *S. aureus* produces more hydrophobic, hydrogen-rich carotenoids during cultivation (Zamudio-Chávez et al. 2023). These carotenoids are insoluble in water and need to be washed repeatedly to eliminate the interference of hydrogen isotopes. Therefore, seven washing cycles effectively eliminate interference from these compounds, thereby revealing the true stable isotope composition of the bacterial powder.

### Key parameters for instrument determination

The last crucial step in this method is to set the important parameters for instrument measurement (step 9). We set multiple TC-EA temperatures (ranging from 1050 to 1450 °C, with an increment of 50 °C), and measured the peak areas of H_2_ and CO, together with the *δ*^2^H and *δ*^18^O values, for both the NB medium and bacterial powders (Fig. S9-S12). Similarly, multiple Flash EA temperatures (ranging from 700 to 950 °C, with an increment of 50 °C) were established, and the peak areas of CO_2_ and N_2_, together with the *δ*^13^C and *δ*^15^N values of the NB medium and bacterial powders, were measured (Fig. S9-S12). 

Specifically, when the TC-EA temperature was below 1350 °C, the H_2_ peak areas and *δ*^2^H values for NB medium (Fig. S9a, S10a), *E. coli* (Fig. S9b, S10b), and *S. aureus* (Fig. S9c, S10c) increased with temperature. However, above 1350 °C, these measured values plateaued. A similar trend was observed for CO peak areas and *δ*^18^O values (Fig. S9d–f, S10d–f). Therefore, 1350 °C was selected as the optimal temperature for the TC-EA. Correspondingly, 800 °C was determined to be the optimal temperature for the EA (Fig. S11, S12).

In summary, key experimental parameters were optimized and validated, which included bacterial incubation duration (19 h), number of washing cycles (7 cycles), pyrolysis temperature (1350 °C), and combustion temperature (800 °C). These well-defined experimental conditions further ensure the accuracy and stability of the generated isotope ratio analysis data. Consequently, this study has established a robust and standardized stable isotope analysis method for bacteria and their culture systems, laying a rigorous technical foundation for bacterial traceability.

### The isotopic relationship between bacteria and their culture water can infer the cultivation region

**Fig. 2:**
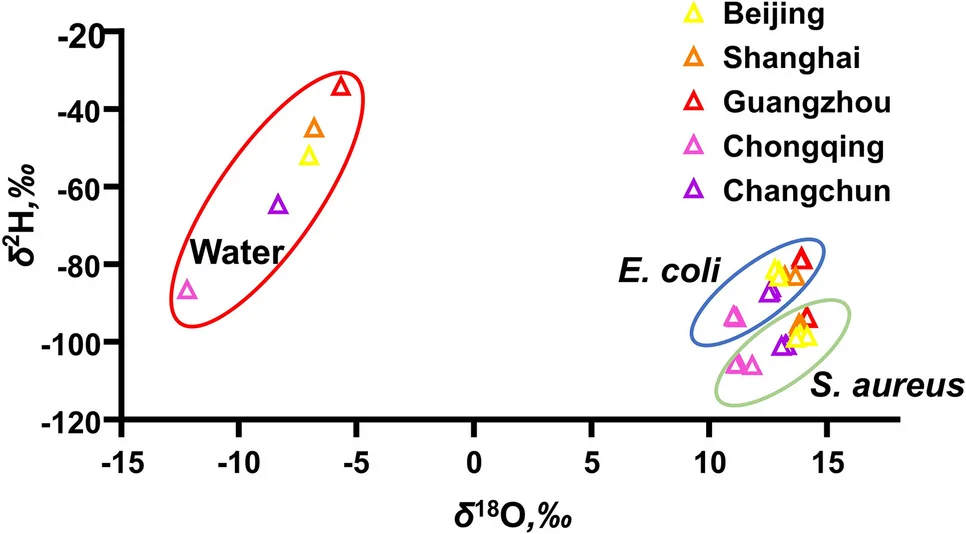
Distribution of hydrogen and oxygen isotope ratios in culture water, *E. coli*, and *S. aureus*

The analysis of water stable isotopes is one of the most powerful tools for establishing a connection between organisms and geographical information (Kreuzer-Martin et al. 2004; Kreuzer-Martin and Jarman 2007). To simulate practical scenarios, complete culture media were prepared using NB medium powder and pure water samples from five cities. These media were divided into three aliquots and used immediately to cultivate *E. coli* and *S. aureus*. The harvested bacteria were then lyophilized, sterilized, and randomly divided into three sub-samples per portion (resulting in *n* = 9 per condition) before final packaging and stable isotope analysis (step 1 in Fig. 1). The *δ*^2^H and *δ*^18^O values of pure water samples and the two types of bacteria were measured (Fig. 2), the result showed that the *δ*^2^H and *δ*^18^O of water samples from five cities were separated pairwise and did not interfere with each other (red circles). This provides a basis for inferring the cultivation location of bacteria using the stable isotope determination of the culture water.

Furthermore, analysis of the bacterial powder relative to the culture water revealed distinct isotope fractionation patterns (Fig. 2). For both *E. coli* and *S. aureus*, the bacterial *δ*^18^O values were increased (ranging from 10‰ to 15‰, blue and green circles in Fig. 2) compared to the water (*δ*^18^O, −12‰ to −5‰; red circles in Fig. 2). In contrast, the bacterial *δ*^2^H values were decreased (ranging from −80‰ to −100‰, blue and green circles in Fig. 2) relative to the water (*δ*^2^H, −30‰ to −90‰; red circles in Fig. 2). This indicates that during growth, both types of bacteria preferentially uptake the heavier oxygen isotope and the lighter hydrogen isotope, resulting in ^18^O enrichment and ^2^H depletion. The extent of this fractionation was slightly more pronounced in *S. aureus* than in *E. coli*.

**Fig. 3:**
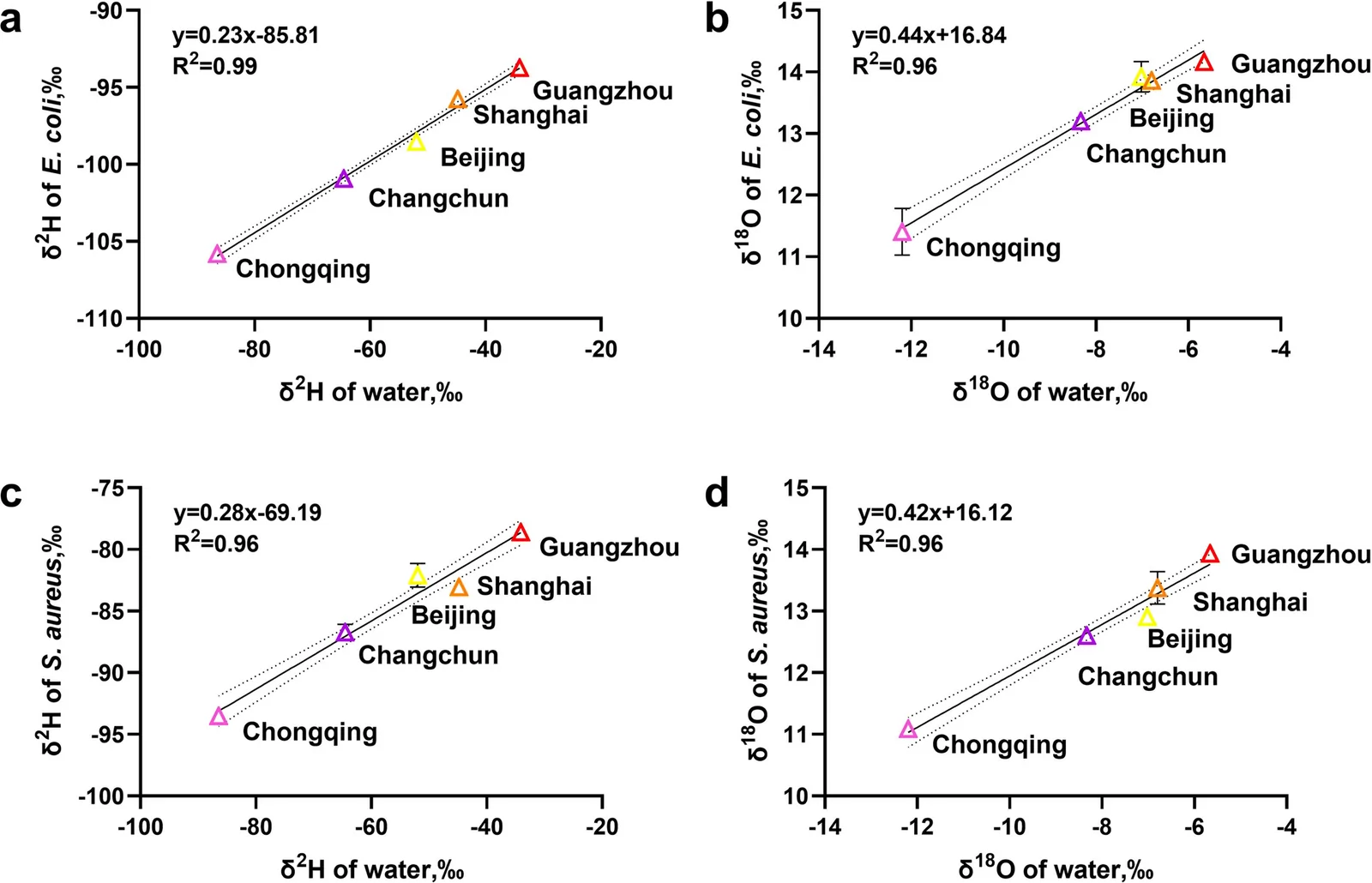
Relationships between hydrogen and oxygen isotope ratios of *E. coli* (**a**, **b**) and *S. aureus* (**c**, **d**) and culture water

Next, to specifically clarify the correlation between the hydrogen and oxygen stable isotope ratios of bacteria and their culture water, scatter plots were drawn using the hydrogen stable isotope ratios of the culture water and those of *E. coli* and *S. aureus* (Fig. 3a, Fig. 3c). Similarly, scatter plots of the oxygen stable isotope ratios of water and the two types of bacteria were also drawn (Fig. 3b, Fig. 3d). Results of linear regression analysis showed that both types of bacteria had a positive correlation with the hydrogen and oxygen stable isotopes of the culture water (Fig. 3a–d, with *R*^2^ ≥ 0.96), indicating that the fractionation effects of the same type of bacteria on the hydrogen and oxygen stable isotopes of water were relatively stable. Further analysis revealed that the slopes of Fig. 3a and Fig. 3c were 0.23 and 0.28, respectively, suggesting that approximately 23% and 28% of the hydrogen atoms in *E. coli* and *S. aureus* during growth originated from the culture water. The slopes of Fig. 3b and Fig. 3d were 0.44 and 0.42, respectively, indicating that about 44% and 42% of the oxygen atoms in the two types of bacteria came from the culture water. Therefore, measuring the hydrogen and oxygen stable isotope ratios in these two types of bacteria can determine the hydrogen and oxygen stable isotope ratios of their culture water, thus enabling the inference of the bacteria’s cultivation location, which has practical implications. Since the stable isotope fractionation effects vary among different bacteria, in actual cases, the established stable isotope analysis method can be used to explore the hydrogen and oxygen stable isotope fractionation patterns of the bacteria involved and then infer the cultivation site of the bacteria.

### Multi-isotope combination discriminant models can infer bacteria’s specific sources

Furthermore, to identify the specific sources of bacteria (such as the cultivation laboratory), we conducted research on the relationship between the stable isotopes of culture media and bacteria. To simulate practical scenarios, in this study, LB, TSB, and NB culture media from different manufacturers were selected, with all media prepared using the same source of water. According to the above method (Fig. 1), bacterial powder of *E. coli* (33 samples total, including 9 from LB medium, 9 from TSB medium, and 15 from NB medium) and *S. aureus* (39 samples total, including 9 from LB medium, 9 from TSB medium, and 21 from NB medium) was prepared. The *δ*^2^H, *δ*^18^O, *δ*^13^C, and *δ*^15^N were measured and statistically analyzed to evaluate the effectiveness of different isotope combinations in differentiating the types of culture media.

**Fig. 4:**
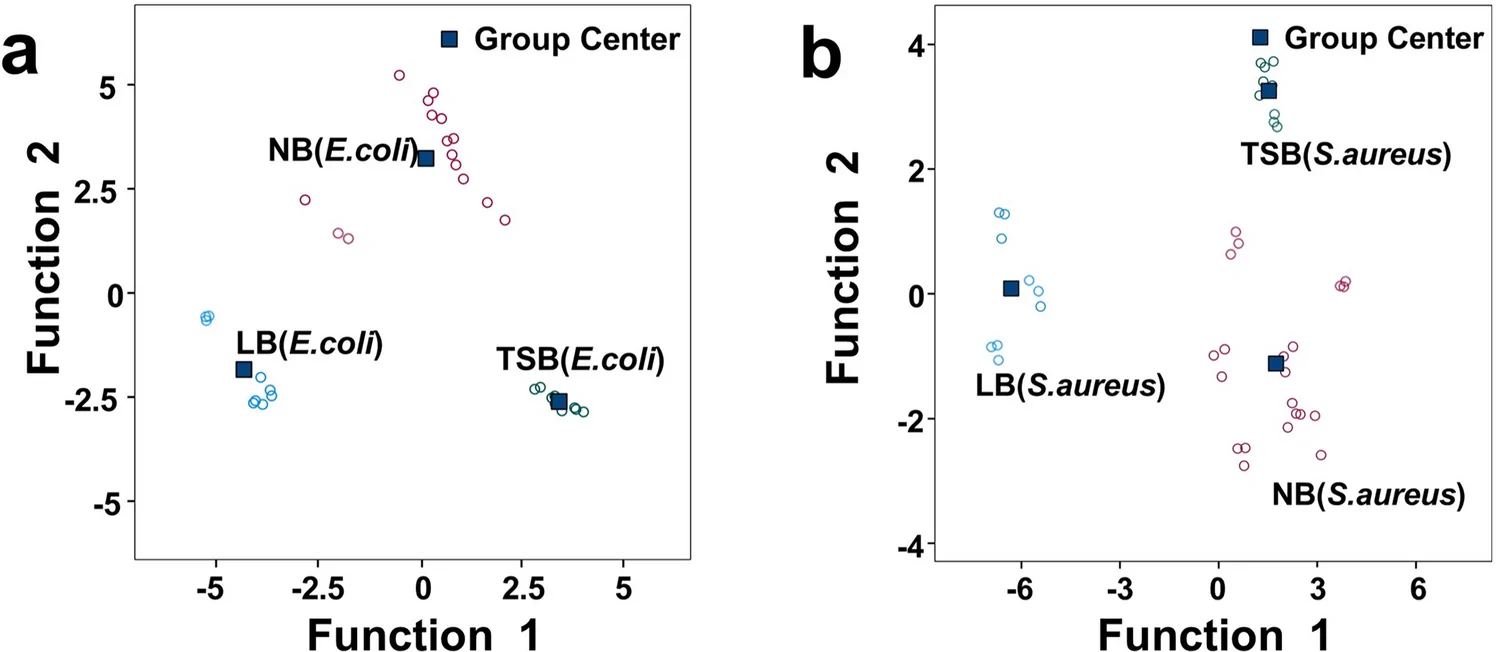
Distribution of *E. coli* (**a**) and *S. aureus* (**b**) on stable isotope discriminant function 1 and function 2

The results showed that two isotope combinations (including the H and O, C and N combinations) led to misclassification in the identification of culture media (Fig. S13). Notably, when a four-isotope combination was adopted, the discrimination of culture medium types was significantly effective (Fig. 4a and Fig. 4b are the characteristic space diagrams of *E. coli* and *S. aureus* respectively), achieving a discrimination accuracy rate of 100% (Table 1).

**Table 1:** Summary of Fisher discriminant functions

	Function	The standardized coefficient				Eigenvalue	Variance contribution rate	Cumulative variance contribution rate	*P*
		*δ*^2^H	*δ*^18^O	*δ*^13^C	*δ*^15^N				
*E. coli*	1	−0.493	0.649	1.262	0.756	8.677	51.4	51.4	< 0.001
	2	1.053	−0.640	−0.333	0.089	8.216	48.6	100.0	< 0.001
*S. aureus*	1	−0.808	−0.221	1.446	1.141	11.887	78.1	78.1	< 0.001
	2	−0.278	0.815	0.239	−0.088	3.324	21.9	100.0	< 0.001

In addition, taking the identification of different culture media for *E. coli* using the four-isotope combination as an example, standardized discriminant functions were constructed. Discriminant function 1 was *Y*_*1*_ = −0.493*X*_*H*_ + 0.649*X*_*O*_ + 1.262*X*_*C*_ + 0.756*X*_*N*_ (with a variance contribution rate of 51.4%, carrying the main original information), and discriminant function 2 was *Y*_*2*_ = 1.053*X*_*H*_ − 0.640*X*_*O*_ − 0.333*X*_*C*_ + 0.089*X*_*N*_ (with a variance contribution rate of 48.6%). The two functions jointly explained 100% of the discriminant information, and the probability values (*P*-values) were both < 0.001, indicating that the model had significant statistical significance. Likewise, the isotope discriminant function model for *S. aureus* also had statistical significance (Table 1). The canonical variable plot (Fig. 4) showed that the group centers corresponding to different culture media were significantly separated in the two-dimensional coordinate system, further confirming the scientific nature of the four-isotope combination discriminant model.

This study clarifies the significant advantages of multi-isotope combinations in the identification of bacterial culture medium types. The multi-isotope combinations can achieve a 100% discrimination accuracy rate, and the constructed statistical model also has significant statistical significance. It can accurately exclude or determine the culture medium, providing a reliable technical method for the associative traceability of bacteria and culture media. This research introduces multi-isotope analysis into the field of bacterial traceability and improves the technical system of microbial stable isotope traceability. In actual cases, such an experimental procedure can be referred to in order to explore the relationship between the bacteria and the culture medium in the case, so as to determine the specific source of the bacteria.

Corresponding to the Fisher discriminant results of function 1 and function 2 presented in Fig. 4

## Discussion

Bacteria, with their remarkable species diversity, can thrive in nearly any extreme environment (Gregory et al. 2024; Seckbach 2009; Wang et al. 2024). However, the outbreak of certain bacteria can trigger severe diseases, threatening public safety and causing significant social panic and economic losses (Clemens et al. 2017). This urgency highlights the urgent need to find an effective method for tracing the origin of bacteria. Stable isotope analysis technology offers a new approach to this end (Nodari et al. 2024).

In this study, we successfully developed a systematic stable isotope analysis method for bacteria and their culture systems. We precisely identified the impact of incubation time, washing cycles, and instrument measurement parameters. Specifically, we determined 19 h as the incubation time for both *E. coli* and *S. aureus*, seven washing cycles, and set specific instrument temperatures (1350 °C for TC-EA, 800 °C for Flash EA). These settings minimized the interference from residual impurities in bacterial cultivation, ensuring the accuracy and reliability of the stable isotope analysis.

Additionally, during the experiment, we explored the sterilization methods and temperatures applicable to bacterial powder. Specifically, we investigated three widely used sterilization approaches: autoclaving, ultraviolet (UV) sterilization, and dry heat sterilization. Autoclaving, which relies on high-temperature steam, interferes with the hydrogen and oxygen stable isotope ratios of the prepared bacterial powder samples, making it unsuitable for subsequent isotope analyses. In contrast, UV sterilization exhibited inadequate sterilization efficacy owing to its limited penetration capacity, failing to achieve thorough decontamination of the bacterial powder matrix. Consequently, dry heat sterilization was selected as the optimal sterilization method, as it does not perturb the stable isotope ratios of bacterial powder samples; a specific treatment regime of 160 °C for 2 h was adopted for this dry heat sterilization process (Rutala and Weber 2008).

We also analyzed several stable isotopes under different TC-EA and EA temperatures. These findings not only lay a rigorous technical foundation for subsequent bacterial traceability studies but also emphasize the importance of considering the unique physiological characteristics of different bacteria. For instance, the distinct behaviors of *E. coli* and *S. aureus* during incubation and washing, due to their different metabolic processes, suggest that in bacterial geographical traceability, attention must be paid to the metabolic differences among species (Bilhère et al. 2009; Maiden et al. 1998). This allows for data correction to ensure result reliability and accuracy.

Due to differences in precipitation, evaporation, etc., across regions, the stable isotope information of water shows distinct regional variations (Craig 1961). Exploring the isotope relationship between bacteria and their culture water is crucial for inferring the cultivation region. We chose two common bacteria, some species of which can cause severe diseases: Gram-negative *E. coli* and Gram-positive *S. aureus* (Gentschev et al. 2002; Yan et al. 2021). By using water from five regions to culture them, we found a significant positive correlation between the hydrogen and oxygen stable isotopes of the bacteria and the culture water. This indicates a consistent isotope fractionation trend in both bacteria, which serves as a reliable indicator for geographical traceability. However, in some regions with similar precipitation and evaporation (Holmes et al. 2024), the hydrogen-oxygen stable isotope ratios of local water may be close, potentially leading to misjudgments. Thus, such factors need to be excluded during result analysis.

To obtain more information about the bacteria’s cultivation location, we explored the relationship between bacteria and their culture media. Employing multi-isotope combination discriminant models to identify the specific sources of bacteria is a major breakthrough. A four-isotope combination (*δ*^2^H, *δ*^18^O, *δ*^13^C, and *δ*^15^N) achieved a 100% accuracy rate in differentiating culture media types, outperforming two-isotope combinations. The construction of statistically significant discriminant functions for both *E. coli* and *S. aureus* validates the model’s effectiveness. This model offers a reliable method for associative traceability of bacteria and culture media, enabling determination of the specific source, such as the cultivation laboratory. Currently, we are delving deeper into bacterial metabolism and using more culture media types to comprehensively verify the discriminant model’s effectiveness (Feng et al. 2018; Schoeler and Caesar 2019).

In conclusion, this study represents a significant advancement by establishing a comprehensive stable isotope analysis method for bacteria and their culture systems. It not only demonstrates the feasibility of using stable isotope analysis to trace bacterial cultivation sites but also lays a groundwork for future research, with broad potential applications in disease control, food safety, and environmental monitoring.

## Supplementary Information

Below is the link to the electronic supplementary material.

ESM 1 (DOCX 592 KB)

## Data Availability

Data is provided within the manuscript or supplementary information files.
