# Supplementary material for: Geographical traceability of bacteria based on a systematic stable isotope analysis method
Source: Appl Microbiol Biotechnol. 2026 Jan 19;110(1):27. doi: 10.1007/s00253-025-13704-x (PMC12819524; doi:10.1007/s00253-025-13704-x)
Supplement: Supplementary file 1 — (DOCX 592 KB) [file 253_2025_13704_MOESM1_ESM.docx]

**Supplementary materials**

**Geographical** **traceability of bacteria based on** **a systematic**

**stable isotope analysis method**

Wei Wang^1,2,3, #^, Bichun Zhao^4, #^, Zhuotong Cai^1,2^, Zhaowei Jie^1,2^, Lin Feng^1,2^, Can Hu^2^, Hongling Guo^2^, Yajun Li^2^, Xianhe Deng^2^, Jun Zhu^2*^, Hongcheng Mei^2*^, Jian Ye^2*^

*1 People’s Public Security University of China, Beijing 100038, China.*

*2 Institute of Forensic Science, Ministry of Public Security of China, Beijing 100038, China.*

*3 Department of public security of Shanxi Province, Shanxi 030001, China.*

*4 Beijing University of Chinese Medicine, Beijing 102488, China.*

*#* These authors contributed equally: Wei Wang, Bichun Zhao.

* **Corresponding author:** Jun Zhu, Hongcheng Mei, Jian Ye.

*Institute of Forensic Science, Ministry of Public Security of China, Beijing 100038, China.*

E-mail address: zhujun001cn@126.com; meihongcheng@163.com; yejian77@126.com.

**
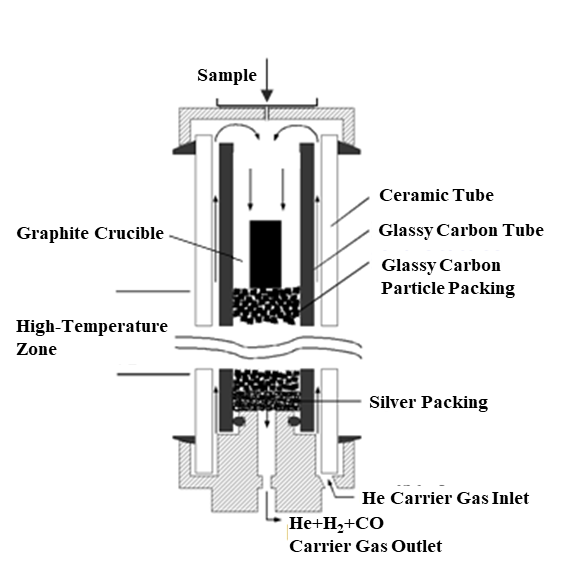
**

Fig. S1 High-Temperature Pyrolysis Module


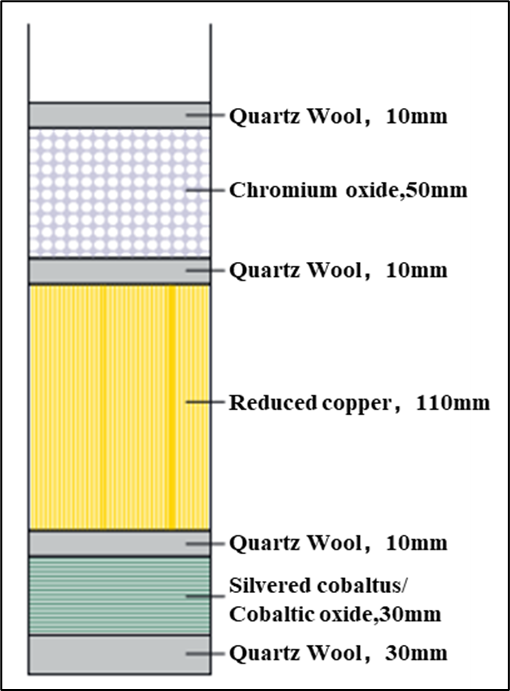


Fig. S2 Dynamic Rapid Combustion Module

**Fig. S3 Growth curve of *E.coli***

Determine the OD620 value of bacteria every 2 - hour interval.

**Fig. S4 Growth curve of** ***S.*** ***aureus***

Determine the OD620 value of bacteria every 2 - hour interval.


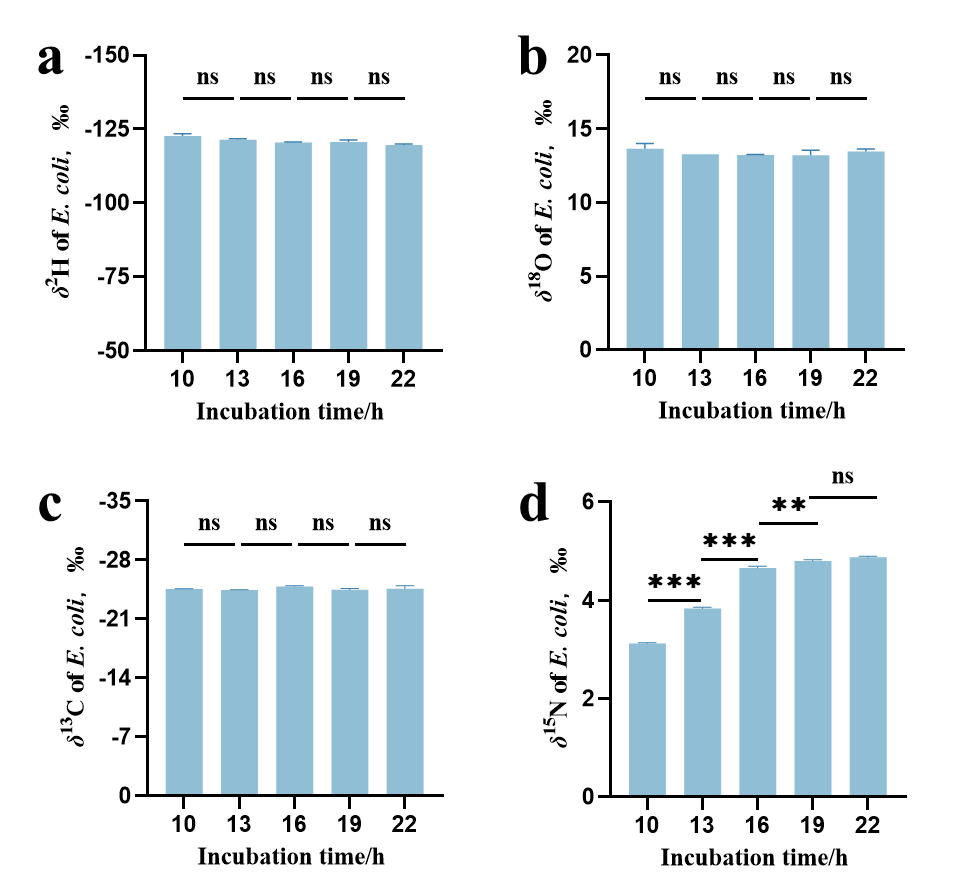


**Fig. S5 Stable isotope measurement results of *E. coli* at different**

**incubation times (10 h, 13 h, 16 h, 19 h, 22 h)**

(a) *δ*^2^H values of *E. coli* at different incubation times. Data are expressed as mean ± standard deviation (SD) (n = 9).

(b) *δ*^18^O values of *E. coli* at different incubation times. Data are expressed as mean ± SD (n = 9).

(c) *δ*^13^C values of *E. coli* at different incubation times. Data are expressed as mean ± SD (n = 9).

(d) *δ*^15^N values of *E. coli* at different incubation times. Data are expressed as mean ± SD (n = 9).

Error bars representing the SD. * indicated *P* < 0.05, ** indicated *P* < 0.01, *** indicated *P* < 0.001, ns indicated *P* > 0.05.


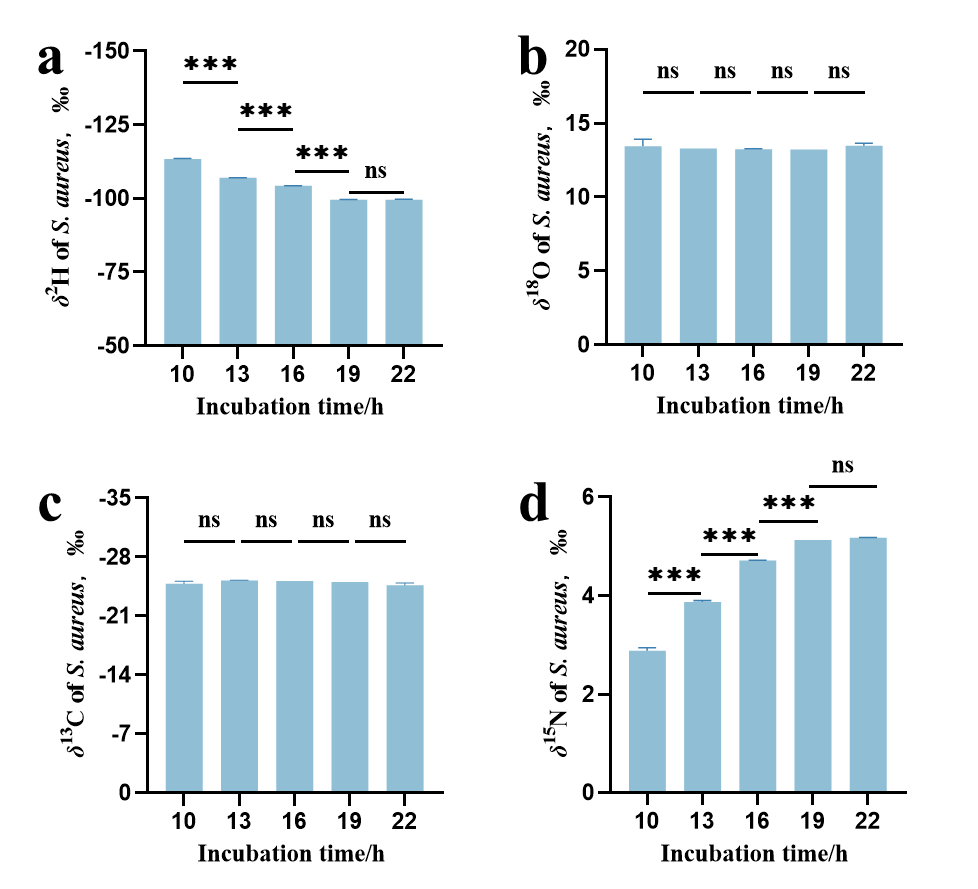


**Fig. S6 Stable isotope measurement results of *S. aureus* at different**

**incubation times (10h, 13h, 16h, 19h, 22 h)**

(a) *δ*^2^H values of *S. aureus* at different incubation times. Data are expressed as mean ±SD (n = 9).

(b) *δ*^18^O values of *S. aureus* at different incubation times. Data are expressed as mean ± SD (n = 9).

(c) *δ*^13^C values of *S. aureus* at different incubation times. Data are expressed as mean ± SD (n = 9).

(d) *δ*^15^N values of *S. aureus* at different incubation times. Data are expressed as mean ± SD (n = 9).

Error bars representing the SD. * indicated *P* < 0.05, ** indicated *P* < 0.01, *** indicated *P* < 0.001, ns indicated *P* > 0.05.


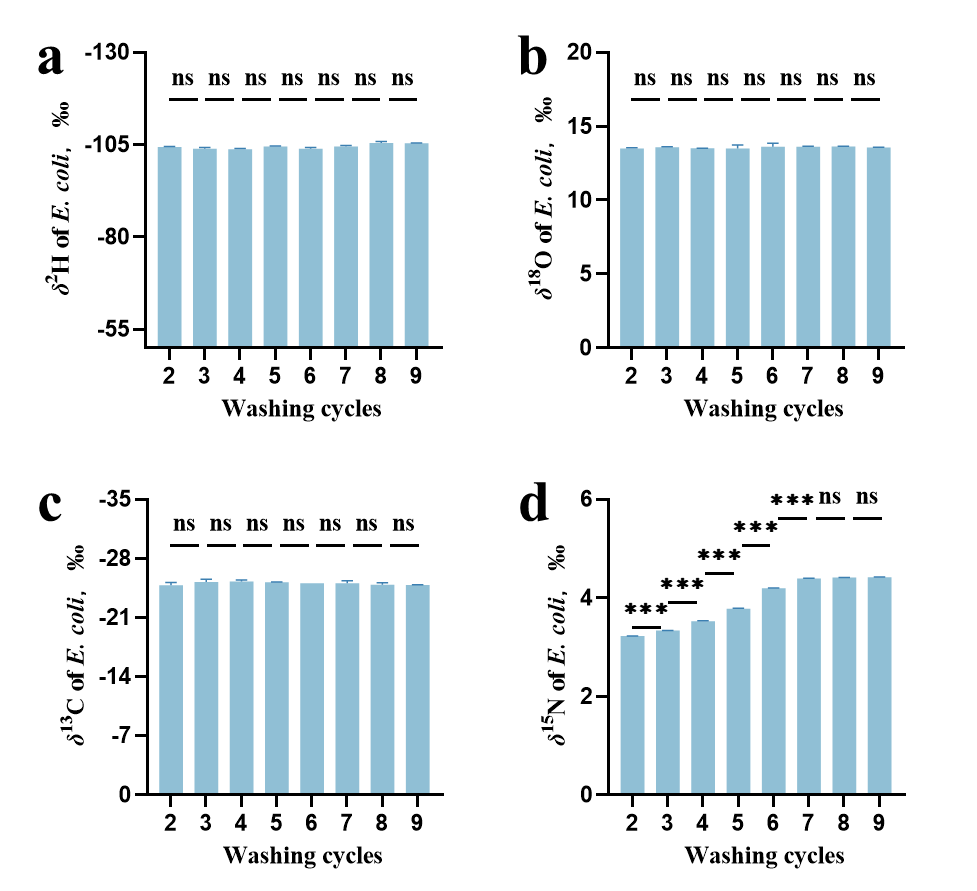


**Fig. S7 Stable isotope measurement results of *E. coli* at different washing cycles**

(a) *δ*^2^H values of *E. coli* at different washing cycles. Data are expressed as mean ±SD (n = 9).

(b) *δ*^18^O values of *E. coli* at different washing cycles. Data are expressed as mean ± SD (n = 9).

(c) *δ*^13^C values of *E. coli* at different washing cycles. Data are expressed as mean ± SD (n = 9).

(d) *δ*^15^N values of *E. coli* at different washing cycles. Data are expressed as mean ± SD (n = 9).

Error bars representing the SD. * indicated *P* < 0.05, ** indicated *P* < 0.01, *** indicated *P* < 0.001, ns indicated *P* > 0.05.


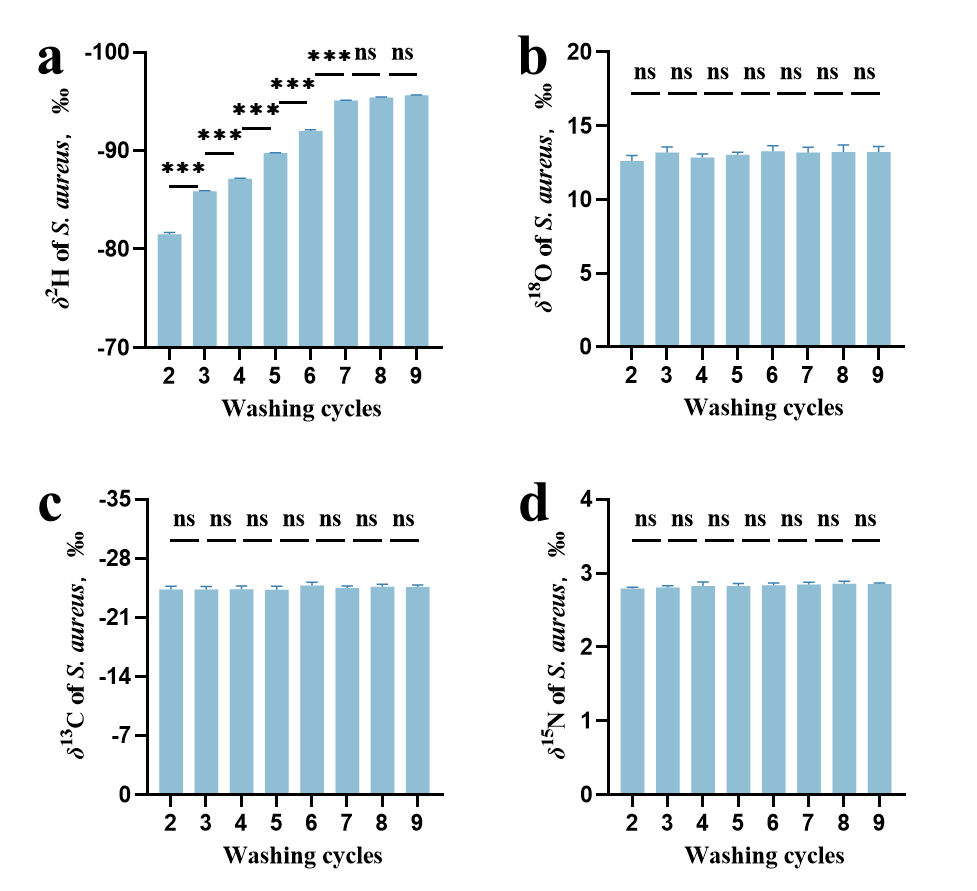


**Fig. S8 Stable isotope measurement results of *S. aureus* at different washing cycles**

(a) *δ*^2^H values of *S. aureus* at different washing cycles. Data are expressed as mean ±SD (n = 9).

(b) *δ*^18^O values of *S. aureus* at different washing cycles. Data are expressed as mean ± SD (n = 9).

(c) *δ*^13^C values of *S. aureus* at different washing cycles. Data are expressed as mean ± SD (n = 9).

(d) *δ*^15^N values of *S. aureus* at different washing cycles. Data are expressed as mean ± SD (n = 9).

Error bars representing the SD. * indicated *P* < 0.05, ** indicated *P* < 0.01, *** indicated *P* < 0.001, ns indicated *P* > 0.05.


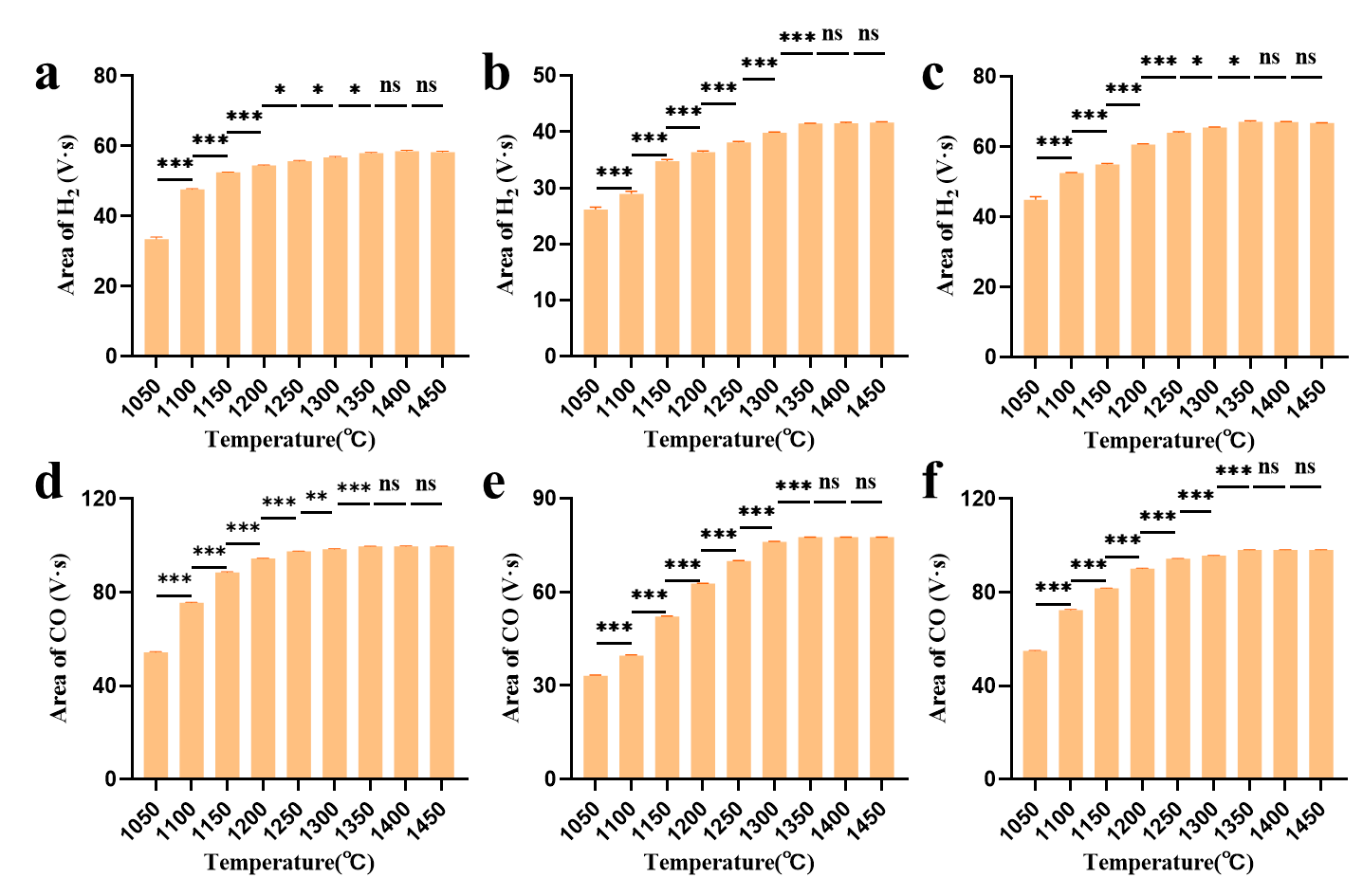


**Fig. S9 Stable isotope measurement results of culture media and bacterial samples**

**at TC-EA temperatures (1050–1450°C)**

(a) Peak area for H_2_ of the culture medium at different TC-EA temperatures. Data are expressed as mean ±SD (n = 9).

(b) Peak area for H_2_ of *E. coli* at different TC-EA temperatures. Data are expressed as mean ±SD (n = 9).

(c) Peak area for H_2_ of *S. aureus* at different TC-EA temperatures. Data are expressed as mean ±SD (n = 9).

(d) Peak area for CO of the culture medium at different TC-EA temperatures. Data are expressed as mean ±SD (n = 9).

(e) Peak area for CO of *E. coli* at different TC-EA temperatures. Data are expressed as mean ±±SD (n = 9).

(f) Peak area for CO of *S. aureus* at different TC-EA temperatures. Data are expressed as mean ±SD (n = 9).

Error bars representing the SD. * indicated *P* < 0.05, ** indicated *P* < 0.01, *** indicated *P* < 0.001, ns indicated *P* > 0.05.

**
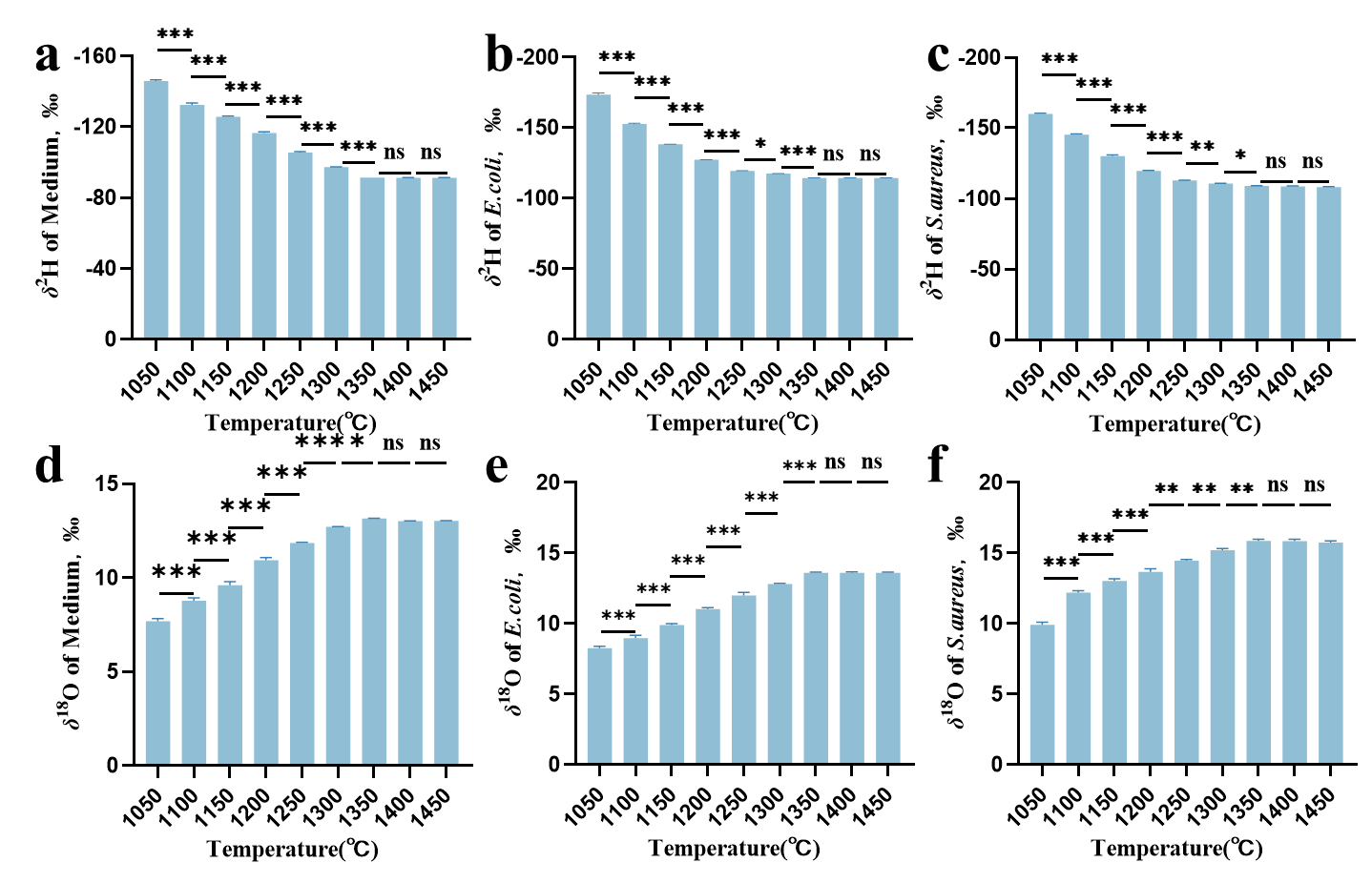
**

**Fig. S10 Stable isotope measurement results of culture media and bacterial samples**

**at TC-EA temperatures (1050–1450°C)**

(a) *δ*^2^H values of the culture medium at different TC-EA temperatures. Data are expressed as mean ±SD (n = 9).

(b) *δ*^2^H values of *E. coli* at different TC-EA temperatures. Data are expressed as mean ±SD (n = 9).

(c) *δ*^2^H values of *S. aureus* at different TC-EA temperatures. Data are expressed as mean ±SD (n = 9).

(d) *δ*^18^O values of the culture medium at different TC-EA temperatures. Data are expressed as mean ±SD (n = 9).

(e) *δ*^18^O values of *E. coli* at different TC-EA temperatures. Data are expressed as mean ±SD (n = 9).

(f) *δ*^18^O values of *S. aureus* at different TC-EA temperatures. Data are expressed as mean ±SD (n = 9).

Error bars representing the SD. * indicated *P* < 0.05, ** indicated *P* < 0.01, *** indicated *P* < 0.001, ns indicated *P* > 0.05.

**
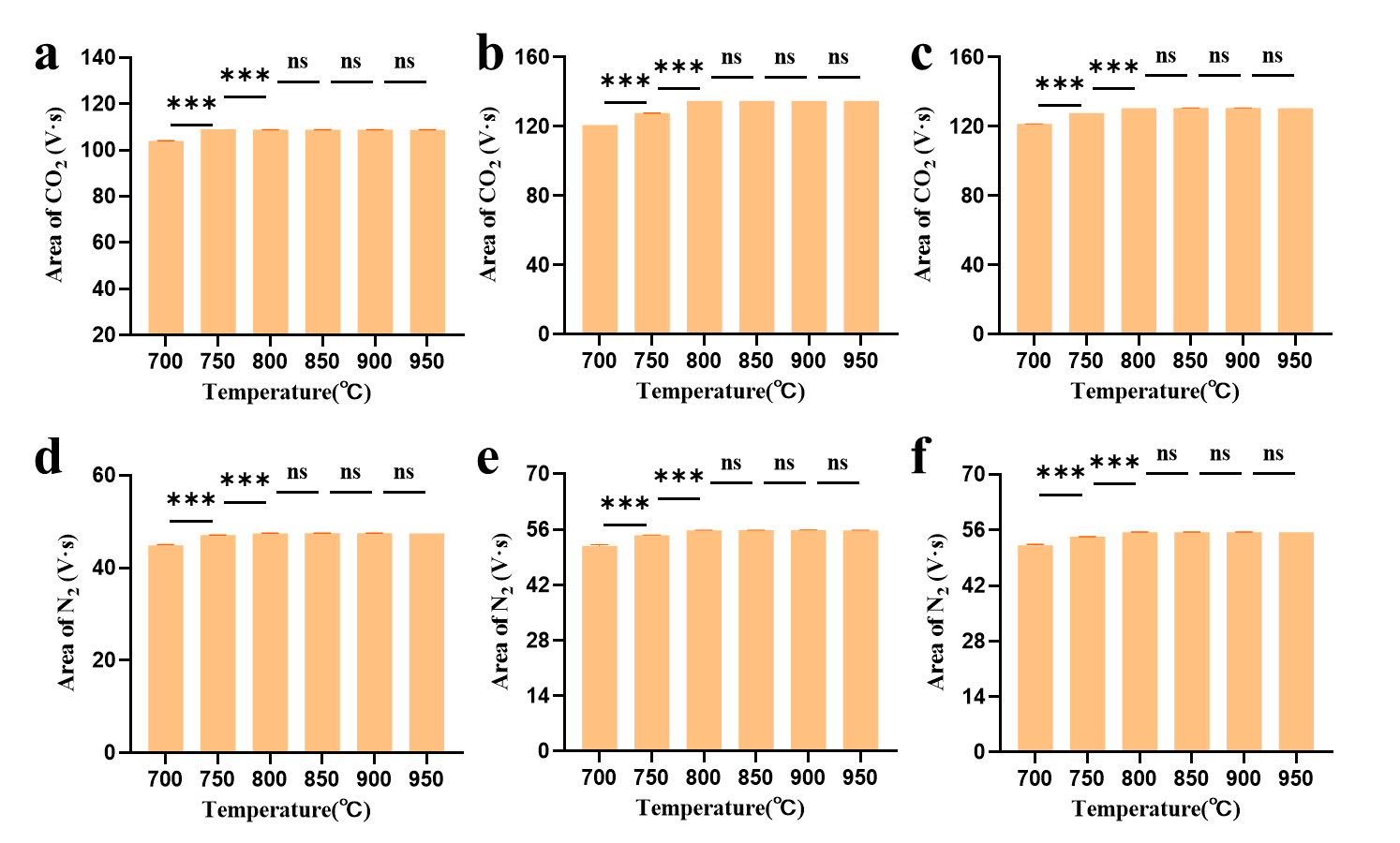
**

**Fig. S11 Stable isotope measurement results of culture media and bacterial samples**

**at EA temperatures** **(700–950°C)**

(a) Peak area for H_2_ of the culture medium at different EA temperatures. Data are expressed as mean ±SD (n = 9).

(b) Peak area for H_2_ of *E. coli* at different EA temperatures. Data are expressed as mean ±SD (n = 9).

(c) Peak area for H_2_ of *S. aureus* at different EA temperatures. Data are expressed as mean ±SD (n = 9).

(d) Peak area for CO of the culture medium at different EA temperatures. Data are expressed as mean ±SD (n = 9).

(e) Peak area for CO of *E. coli* at different EA temperatures. Data are expressed as mean ±SD (n = 9).

(f) Peak area for CO of *S. aureus* at different EA temperatures. Data are expressed as mean ±SD (n = 9).

Error bars representing the SD. * indicated *P* < 0.05, ** indicated *P* < 0.01, *** indicated *P* < 0.001, ns indicated *P* > 0.05.

**
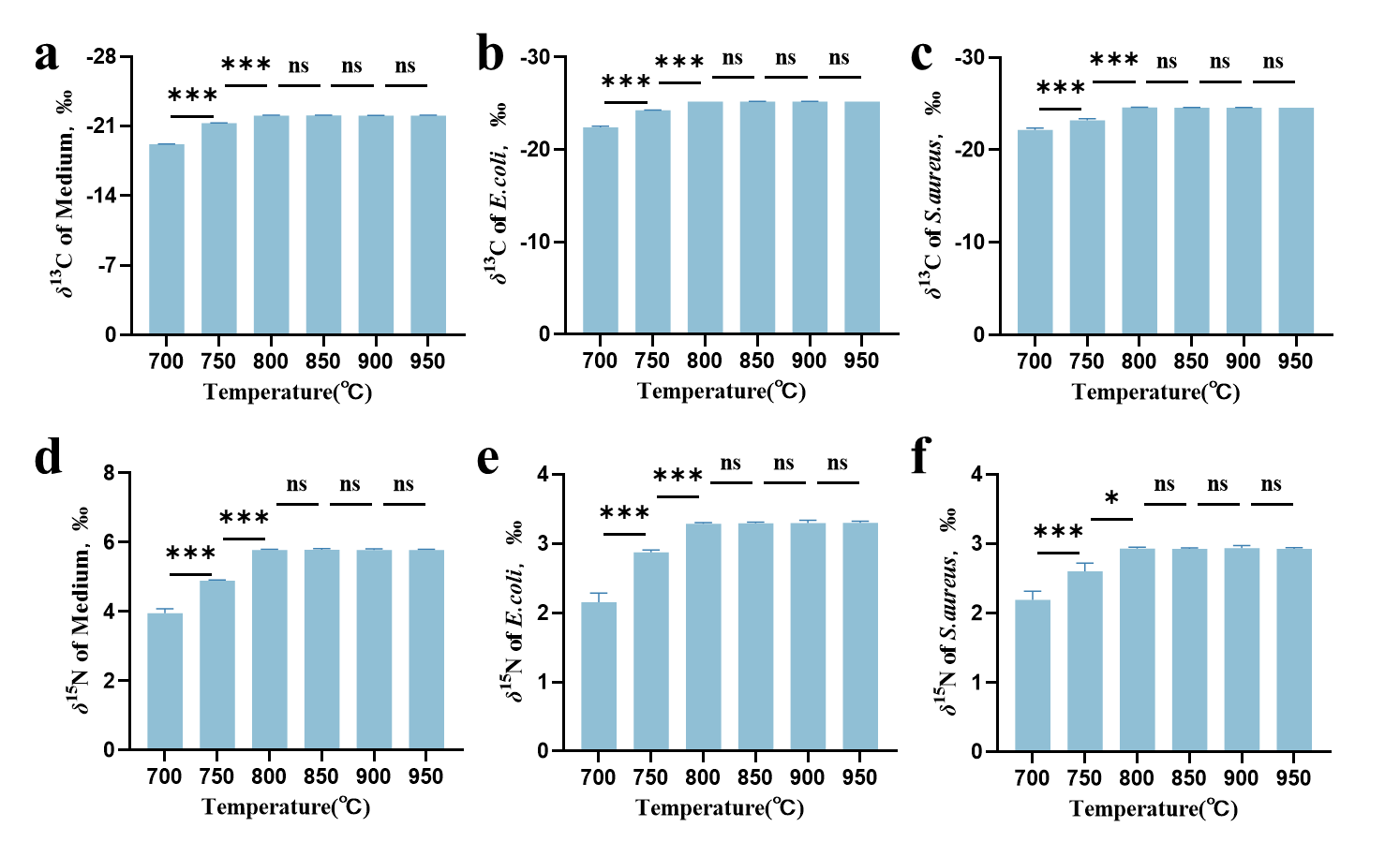
**

**Fig. S12 Stable isotope measurement results of culture media and bacterial samples**

**at EA temperatures (700–950°C)**

(a) *δ*^2^H values of the culture medium at different EA temperatures. Data are expressed as mean ±SD (n = 9).

(b) *δ*^2^H values of *E. coli* at different EA temperatures. Data are expressed as mean ±SD (n = 9).

(c) *δ*^2^H values of *S. aureus* at different EA temperatures. Data are expressed as mean ±SD (n = 9).

(d) *δ*^18^O values of the culture medium at different EA temperatures. Data are expressed as mean ±SD (n = 9).

(e) *δ*^18^O values of *E. coli* at different EA temperatures. Data are expressed as mean ±SD (n = 9).

(f) *δ*^18^O values of *S. aureus* at different EA temperatures. Data are expressed as mean ±SD (n = 9).

Error bars representing the SD. * indicated *P* < 0.05, ** indicated *P* < 0.01, *** indicated *P* < 0.001, ns indicated *P* > 0.05.

**
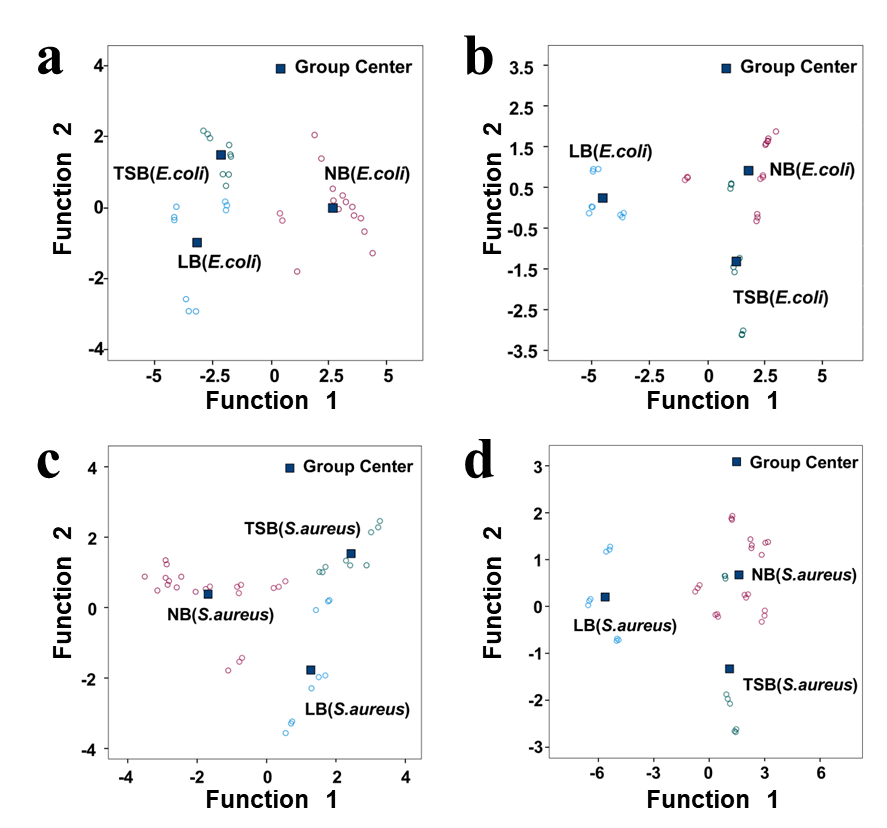
**

**Fig. S13 Distribution of bacteria on Stable Isotope Discriminant**

**Function 1 and Function 2**

(a) H and O stable isotope combinations in discriminating culture medium types for *E. coli*

(b) C and N stable isotope combinations in discriminating culture medium types for *E. coli*

(c) H and O stable isotope combinations in discriminating culture medium types for *S. aureus*

(d) C and N stable isotope combinations in discriminating culture medium types for *S. aureus*
